# Plexin-B2 promotes invasive growth of malignant glioma

**DOI:** 10.18632/oncotarget.3421

**Published:** 2015-01-31

**Authors:** Audrey P. Le, Yong Huang, Sandeep C. Pingle, Santosh Kesari, Huaien Wang, Raymund L. Yong, Hongyan Zou, Roland H. Friedel

**Affiliations:** ^1^ Department of Neuroscience, Friedman Brain Institute, Icahn School of Medicine at Mount Sinai, New York, NY, USA; ^2^ Department of Neurosurgery, Icahn School of Medicine at Mount Sinai, New York, NY, USA; ^3^ Comprehensive Brain Tumor Program, Icahn School of Medicine at Mount Sinai, New York, NY, USA; ^4^ Translational Neuro-Oncology Laboratories, Moores UCSD Cancer Center and Department of Neurosciences, La Jolla, CA, USA

**Keywords:** plexin, semaphorin, glioma invasion, tumor vasculature

## Abstract

Invasive growth is a major determinant of the high lethality of malignant gliomas. Plexin-B2, an axon guidance receptor important for mediating neural progenitor cell migration during development, is upregulated in gliomas, but its function therein remains poorly understood. Combining bioinformatic analyses, immunoblotting and immunohistochemistry of patient samples, we demonstrate that Plexin-B2 is consistently upregulated in all types of human gliomas and that its expression levels correlate with glioma grade and poor survival. Activation of Plexin-B2 by Sema4C ligand in glioblastoma cells induced actin-based cytoskeletal dynamics and invasive migration *in vitro*. This proinvasive effect was associated with activation of the cell motility mediators RhoA and Rac1. Furthermore, costimulation of Plexin-B2 and the receptor tyrosine kinase Met led to synergistic Met phosphorylation. In intracranial glioblastoma transplants, Plexin-B2 knockdown hindered invasive growth and perivascular spreading, and resulted in decreased tumor vascularity. Our results demonstrate that Plexin-B2 promotes glioma invasion and vascularization, and they identify Plexin-B2 as a potential novel prognostic marker for glioma malignancy. Targeting the Plexin-B2 pathway may represent a novel therapeutic approach to curtail invasive growth of glioblastoma.

## INTRODUCTION

Gliomas are primary brain tumors that are among the most lethal forms of cancer [1, 2]. Glioblastoma, the most malignant form of glioma, carries a dismal prognosis of less than 2 year median survival [3]. A major determinant of the high lethality of glioblastoma is its diffuse invasion into healthy brain tissue, thus precluding complete surgical resection. Although genomic analyses have unraveled molecular pathways that drive gliomagenesis [4], the mechanisms by which glioma cells infiltrate healthy brain tissue remain largely unknown, and the signaling molecules that regulate the cell-cell or cell-matrix interactions between glioma and the tumor microenvironment remain poorly defined. A better molecular understanding of glioma invasion is needed to develop therapeutic approaches to curb glioma invasion.

Recent studies suggest that glioma cells actively migrate through the tortuous extracellular spaces of the brain, in much the same way as embryonic neurons and glia cells migrate along preferred extracellular routes in the developing brain [5]. In this regard, glioma cells retain much of their neural origin, and may thus utilize similar signaling pathways as in development. Semaphorins and plexins are ligand/receptor families originally identified as axon guidance molecules in neurodevelopment, but they are increasingly recognized for playing also important roles in tumor growth, migration, metastasis, and vascularization [6, 7]. Several members of the semaphorin and plexin families are highly expressed in gliomas [8, 9], but direct evidence for their engagement in the invasive growth of glioma is lacking.

Here, we focused on the function of Plexin-B2 in the invasive growth of glioma. Among the three Plexin-B receptors (B1-B3), Plexin-B2 is an apparent candidate to play a critical role in glioma growth and invasion, because it was originally cloned as a gene highly upregulated in malignant brain tumors [10]. In addition, recent analyses of glioma patient samples identified Plexin-B2 as a potential biomarker for high-grade glioma [11]. Furthermore, previous studies have established Plexin-B2 as an important regulator for migration of both embryonic and adult neural precursor cells [12-15], but whether Plexin-B2 plays a similar role in glioma invasion is unclear. Plexin-B receptors bind to Semaphorin-4 ligands, a family of six transmembrane Semaphorins (Sema4A-4D, 4F and 4G), but the binding specificities are not yet clearly defined and likely promiscuous. The signaling partners and downstream effectors of the Plexin-B2 pathway in glioma are also not understood. In non-CNS tumors, Plexin-B1 is the best-studied Plexin-B. Upon activation by Sema4D, Plexin-B1 can interact with the receptor tyrosine kinase (RTK) Met to promote migration in several carcinoma cell lines [16, 17], and similar synergistic interaction of Plexin-B1 with ErbB2 has been reported in breast cancer cells [18, 19].

In this study, we show that Plexin-B2 is consistently upregulated in human gliomas and that its expression levels correlate with glioma grade and poor survival. We demonstrate that Plexin-B2 activation changes the actin cytoskeleton and promotes migration of glioma cells. Furthermore, we identify RhoA and Rac1 GTPases as downstream effectors of Plexin-B2 and reveal a synergistic activity of Plexin-B2 and Met in Met phosphorylation. Plexin-B2 knockdown hinders invasive migration of glioma cells and perturbs perivascular spreading. Together, our studies support a role of Plexin-B2 as a potential prognostic biomarker and a novel drug target for high-grade glioma.

## RESULTS

### Plexin-B2 is upregulated in glioma

**Figure 1:**
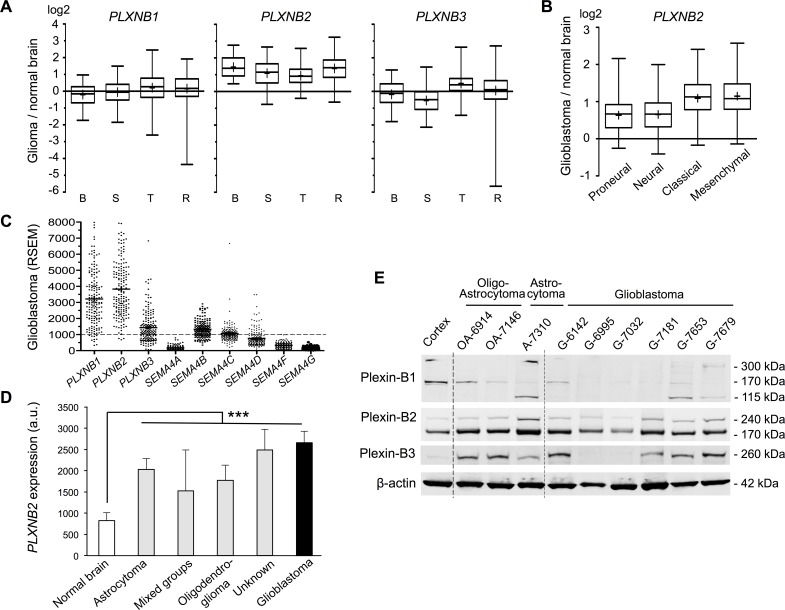
Plexin-B2 is upregulated in glioma A) Relative Plexin-B gene expressions in glioma vs. normal brain from microarray data of four patient studies (B, Bredel et al. (n=49); S, Sun et al. (n=49); T, TCGA (n=424); R, Rembrandt (n=454)). Whiskers show top and bottom quartiles. *PLXNB2* was upregulated in all four glioma studies. B) Relative *PLXNB2* expression in four molecular subtypes of glioblastoma (TCGA dataset; proneural, n=113; neural, n=70; classical, n=112; mesenchymal, n=128). C) Abundance of Plexin-B and Sema4 mRNA in TCGA glioblastoma samples (n=169), shown as RSEM counts (upper quartile boundary normalized to 1000). *PLXNB2* exhibited the highest mean abundance level overall, and *SEMA4B*, *4C* and *4D* exhibited higher levels than other Sema4s. D) *PLXNB2* mean expression is significantly increased in all WHO glioma types (NCI/Rembrandt patient cohort; n=454; probeset 208890_s_at) and highest in glioblastoma (*p*<0.001). E) Western blot of nine surgical samples from glioma patients reveals robust Plexin-B2 protein expression (170 kDa band is the processed, mature form of Plexin-B2). Normal human cortex served as control. Plexin-B1 and -B3 protein are variably expressed among glioma samples.

To investigate Plexin-B and Sema4 gene expression changes in gliomas vs. normal brain, we first analyzed microarray data from four different glioma patient sets. Notably, *PLXNB2* expression was on average upregulated by more than 2-fold in all four patient sets (Fig. 1A). In contrast, *PLXNB1* and -*B3* expression levels were highly variable between glioma samples, and on average not significantly different from levels in normal brain. We then analyzed expression changes of Plexin-B genes in the four transcriptional subtypes of glioblastoma [4]. In the TCGA glioblastoma samples, *PLXNB2* expression was on average upregulated by more than 2.5-fold in classical and mesenchymal subtypes, and more than 1.5-fold in proneural and neural subtypes, while the expression levels of *PLXNB1* and *-B3* were largely similar between subtypes (Fig. 1B; Fig. S1B). Among the Sema4 genes, *SEMA4B* and *SEMA4C* showed mild expression increase in gliomas and glioblastoma subtypes, while the other Sema4s appeared mainly unchanged or downregulated (Fig. S1).

To assess the abundance of Plexin-B and Sema4 transcripts in glioblastoma, we surveyed TCGA RNAseq data, which provides quantitative expression levels as RSEM normalized read counts (counts >1000 define the top quartile of genes). All three Plexin-Bs were highly expressed, with *PLXNB2* displaying highest levels on average. Of the Sema4s, *SEMA4B*, *4C*, and *4D* were expressed at robust levels, whereas the other Sema4s were expressed at lower levels (Fig. 1C).

We next analyzed the NCI Rembrandt data for *PLXNB2* expression among different WHO glioma types. *PLXNB2* expression was increased in all glioma types over normal brain, including oligodendroglioma and astrocytoma (grade II-III), and the highest expression levels were found glioblastoma (grade IV) (Fig. 1D), indicating a correlation of *PLXNB2* expression level and glioma grade.

We also confirmed protein expression of Plexin-Bs in a set of surgical samples from glioma patients by Western blot analysis. Plexin-B2 protein was consistently detected at robust levels in all glioma samples; by contrast, Plexin-B1 and -B3 protein levels were highly variable between samples (Fig. 1E).

### Expression of Plexin-B2 in human glioma correlates with shorter survival

**Figure 2:**
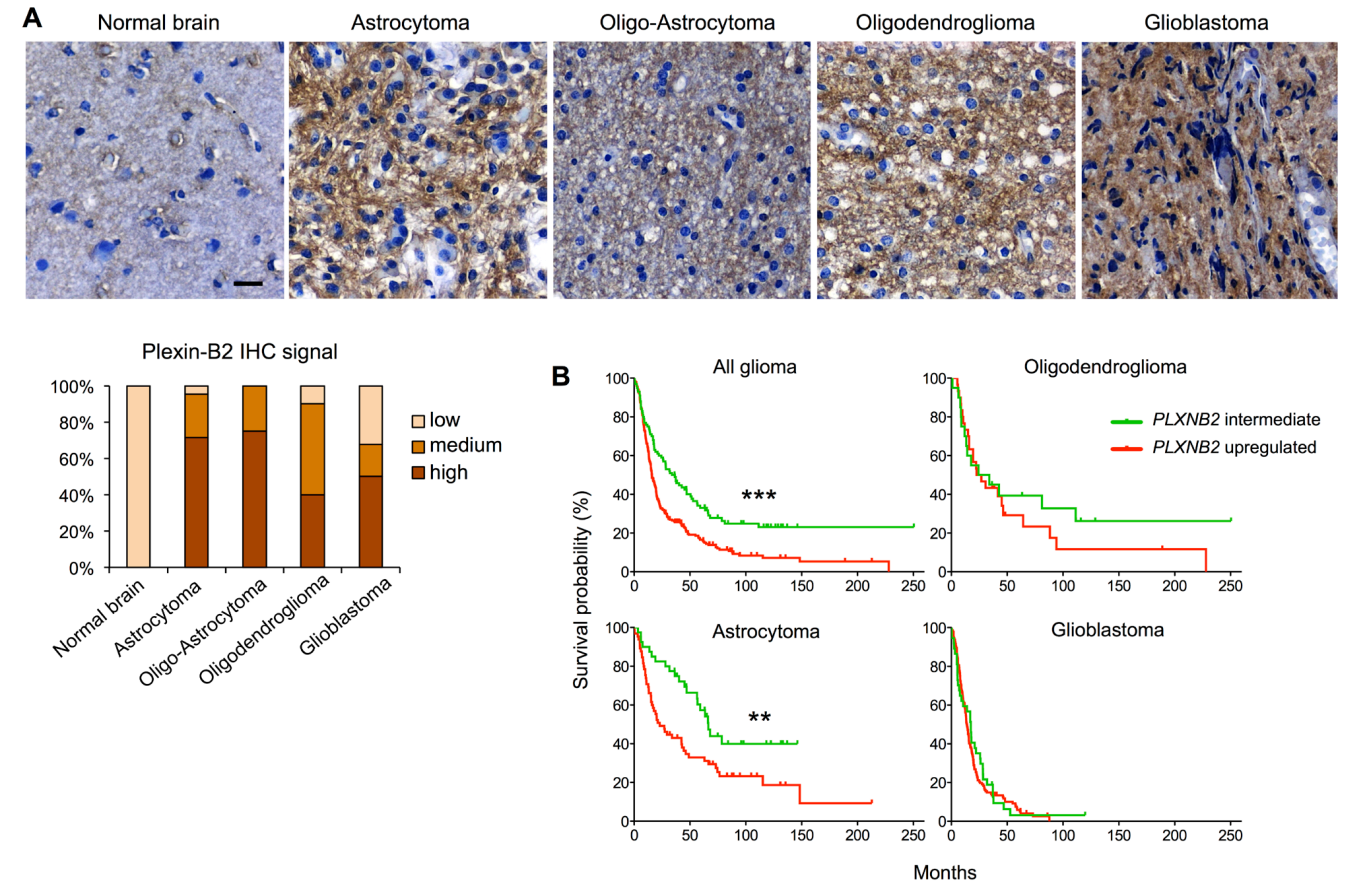
Plexin-B2 expression in glioma correlates with survival A) Representative images of DAB immunolabeling for Plexin-B2 in normal brain and glioma tissues (n=69). Bar graph summarizes scored signal intensities in different glioma samples (“low” refers to Plexin-B2 immunointensity as found in normal brain). Scale bar: 20 μm. B) Kaplan-Meier survival curves of NCI/Rembrandt glioma patient cohorts. Upregulated Plexin-B2 expression (>2-fold of average normal brain) corresponded with shorter survival time of all glioma patients combined (*p*<10^−4^, see Table S1 for details). Upregulated Plexin-B2 expression correlated with shorter survival in astrocytoma patients (*p*<10^−2^), and to a lesser degree in oligodendroglioma and glioblastoma patients.

We next examined Plexin-B2 protein expression by immunohistochemistry on human glioma tissue microarray cores. Using normal brain tissue as a baseline reference, we found elevated Plexin-B2 protein expression in the vast majority of the examined glioma specimens (Fig. 2A).

To explore the clinical significance of Plexin-B2 upregulation in glioma, we performed Kaplan-Meier survival analyses with the NCI/Rembrandt data platform (Fig. 2B and Table S1). Upregulated *PLXNB2* expression (defined as >2-fold above normal level) correlated with shorter median survival (16.0 months in *PLXNB2* upregulated cohort vs. 32.2 months in *PLXNB2* intermediate cohort; *p*<10^−4^). Correspondingly, the 5-year survival rate of glioma patients in the *PLXNB2* upregulated cohort was below 20%, while it was above 40% for patients with intermediate *PLXNB2* levels.

When stratified for glioma types (Fig. 2B and Table S1), astrocytoma patients with upregulated *PLXNB2* exhibited a significantly shorter median survival than those with intermediate level (23.0 vs. 58.2 months, *p*<10^−2^). Oligodendroglioma patients with upregulated *PLXNB2* also exhibited a shorter median survival than those with intermediate level (24.9 vs. 29.3 months). Similarly, for glioblastoma patients, median survival time for the *PLXNB2* upregulated cohort was shorter than that of the intermediate cohort (13.9 vs. 17.5 months). The survival differences for the latter two glioma types did not reach statistical significance, possibly due to small sample sizes for glioma with low Plexin-B2 expression. Notably, the Rembrandt platform stratified gliomas into only *PLXNB2* upregulated or intermediate groups, and did not include a *PLXNB2* downregulated group (defined as <2-fold below normal), reflecting high prevalence of Plexin-B2 upregulation in gliomas. Another possibility to consider is that in glioblastoma, which is the most malignant type of glioma, tumor cells may have acquired multiple mutations that provide mechanistic substitutes for high Plexin-B2 expression. In addition, we also analyzed survival probabilities of the four TCGA molecular subtypes of glioblastoma in relation to Plexin-B2 upregulation (Fig. S2A), which revealed no statistically significant survival differences when standard parameters were applied (>2-fold as threshold for upregulation). However, when we applied a lower threshold for upregulation (>1.25-fold), the proneural subtype showed a statistically significant shorter median survival in the Plexin-B2 upregulated group (Fig. S2B), suggesting that a slight elevation of Plexin-B2 expression may be sufficient to increase the malignant potency of gliomas.

In sum, the patient survival data suggest that Plexin-B2 upregulation in glioma correlates with a poorer patient survival outlook. In contrast, our analyses did not reveal correlations of Plexin-B1 expression levels and glioma grade/type or patient survival, and only weak correlations for Plexin-B3 with lower Plexin-B3 levels corresponding to worse survival (Fig. S3).

### Plexin-B2 regulates actin-based cell morphology in glioma cells

**Figure 3:**
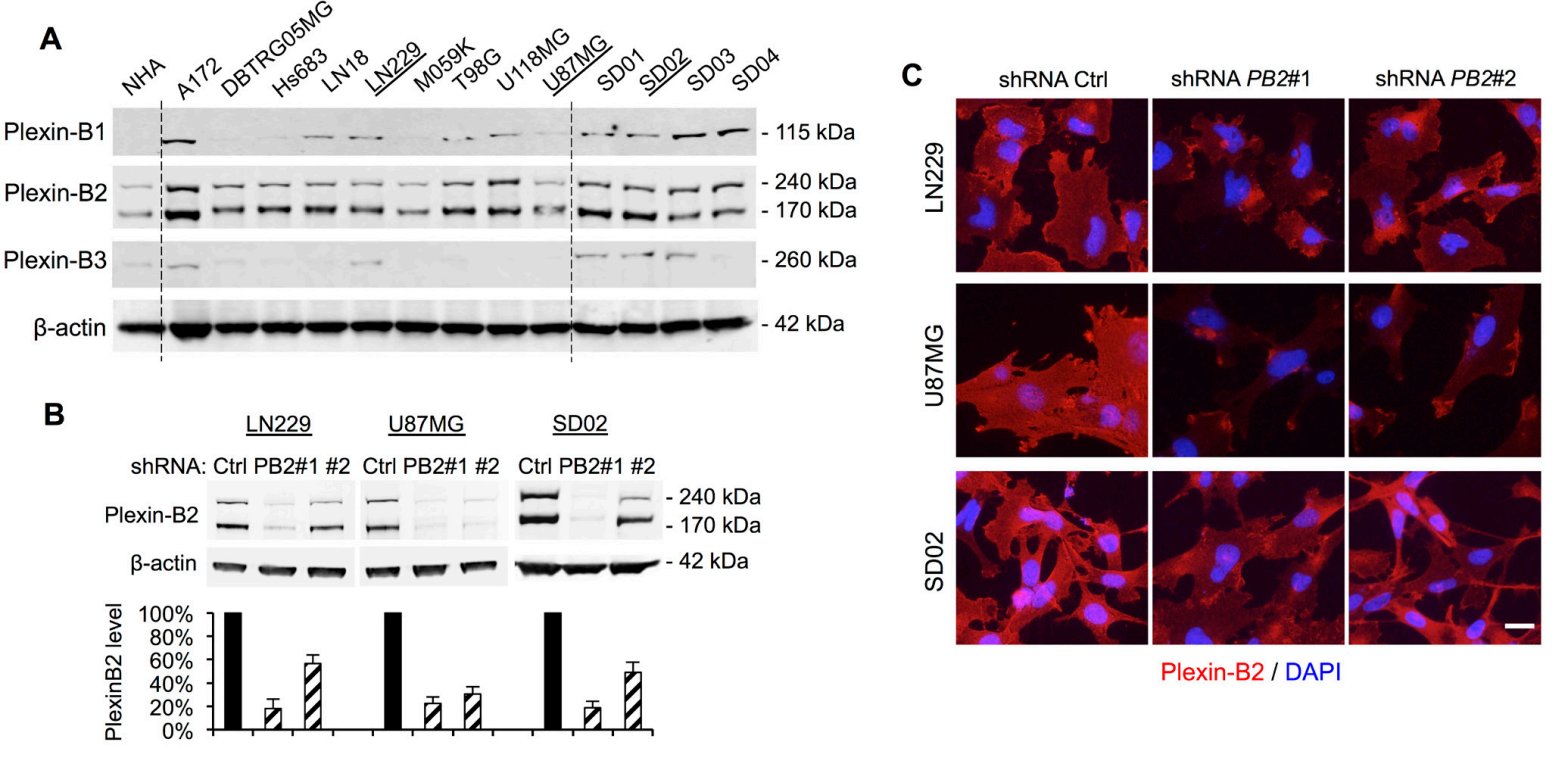
Plexin-B2 expression in glioma cell lines and shRNA knockdown A) Western blot of nine human ATTC glioma cell lines and four glioblastoma stem cell lines (GSC) reveals robust Plexin-B2 protein expression. Normal human astrocytes (NHA) served as control. Plexin-B1 and -B3 are expressed at variable levels in ATCC glioma cell lines and in GSC. B) Stable knockdown of Plexin-B2 with two lentiviral shRNA vectors in LN229, U87MG, and SD02 lines, as measured by Western blot quantification. C) Immunocytochemistry for Plexin-B2 in LN229, U87MG, and SD02 cells confirms reduced Plexin-B2 expression in knockdown lines. Scale bar: 20 μm.

To investigate the mechanisms underlying the link between Plexin-B2 expression and glioma malignancy, we turned to human glioma cells to study Plexin-B2′s roles in mediating cellular processes. We first surveyed Plexin-B protein expression by Western blot in 9 ATTC high-grade glioma lines cultivated in standard serum-containing media and 4 glioblastoma patient lines established in defined neural stem cell media under neurosphere conditions (Fig. 3A). Of note, the latter consist of tumor-propagating cells (also termed glioma stem cells, GSC), with stem-like features such as self-renewal *in vitro* and tumorigenicity *in vivo*. We found that Plexin-B2, but not -B1 or -B3, was consistently detectable in all glioma cell lines examined (Fig. 3A), in line with our findings in patient glioma specimens (see Fig. 1E).

To determine the function of Plexin-B2 in glioma cells, we focused on the ATTC lines LN229 and U87MG and the GSC line SD02, and generated stable knockdown lines using lentiviral shRNA vectors. Western blot and immunocytochemistry confirmed Plexin-B2 knockdown with two different Plexin-B2 shRNA vectors to levels between 15% and 55% of control shRNA lines (Fig. 3B, C).

**Figure 4:**
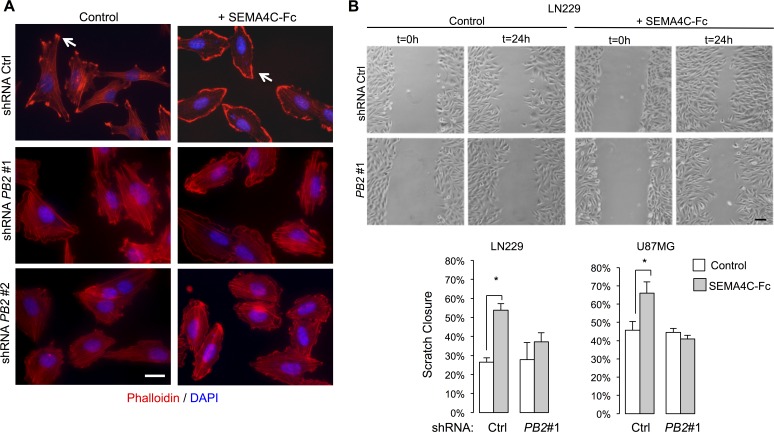
Plexin-B2 activation induces cytoskeletal dynamics in glioma cells A) LN229 cells were stimulated with SEMA4C-Fc or control supernatant for 5 min. Phalloidin staining reveals reduced filamentous actin stress fibers and formation of membrane ruffle following SEMA4C-Fc stimulation in control but less so in Plexin-B2 shRNA knockdown cells. Scale bar: 20 μm. B) Scratch wound assay images of LN229 cells stimulated with either SEMA4C-Fc or control supernatant for 24 hours. Note increased wound closure in control but not in Plexin-B2 shRNA cells with SEMA4C-Fc stimulation. Quantification shows results for LN229 and U87MG cells (n=3; *p*<0.05). Scale bar: 100 μm.

Regulation of the actin cytoskeleton is a major aspect of Plexin function in fibroblasts and neurons [24]. We therefore first examined cytoskeletal effects of Plexin-B2 activation by Sema4C, a ligand for Plexin-B2 in neurodevelopment [14]. Notably, Sema4C is also expressed in gliomas (Fig. 1C; S1; S4). Under control conditions, phalloidin staining of LN229 cells highlighted actin stress fibers traversing cell bodies (Fig. 4A), and immunolabeling for Vinculin, a component of the focal adhesion complex, labeled associated focal adhesions (Fig. S5A). Upon stimulation with recombinant human SEMA4C-Fc, tumor cells displayed a marked reduction in actin stress fibers and focal adhesions, and assumed a more rounded morphology with membrane ruffles formation at cell edges (Fig. 4A; S5A). These effects are reminiscent of the classic lamellipodia formation in 3T3 cells upon Rac1 activation [25]. The reduction in actin stress fibers and the increased formation of membrane ruffles by SEMA4C-Fc stimulation were strongly attenuated in Plexin-B2 shRNA knockdown lines, indicating that the Sema4C-triggered actin dynamics in glioma cells are mediated through Plexin-B2 (Fig. 4A; S5A).

### Plexin-B2 promotes mobility and invasiveness of glioma cells *in vitro*

Next, we examined the effect of Plexin-B2 activation on the migration of glioma cells in a scratch wound assay. In LN229 and U87MG cells, SEMA4C-Fc stimulation resulted in increased wound closure compared to control conditions (Fig. 4B). Importantly, the effect of SEMA4C-Fc stimulation was strongly reduced in Plexin-B2 knockdown cells, demonstrating that the promigratory effect is mediated through Plexin-B2 (Fig. 4B). To rule out a potentially confounding effect from a reduced cell proliferation rate, we confirmed that Plexin-B2 knockdown did not significantly affect the proliferation rates of glioma cells *in vitro* (Fig. S5B).

We also utilized Matrigel transwell assays to measure the effect of Plexin-B2 activation on the invasive migration capacity of glioma cells to penetrate an extracellular matrix substrate. SEMA4C-Fc stimulation of LN229 glioma cells led to a 3-fold increase in the invasive migration relative to control (Fig. S5C). This proinvasive effect of SEMA4C-Fc was significantly reduced but not completely abrogated by Plexin-B2 knockdown. This suggests possible residual Plexin-B2 activity under knockdown conditions or other compensatory mechanisms.

### Plexin-B2 activation regulates Rho family GTPases and RTK signaling

**Figure 5:**
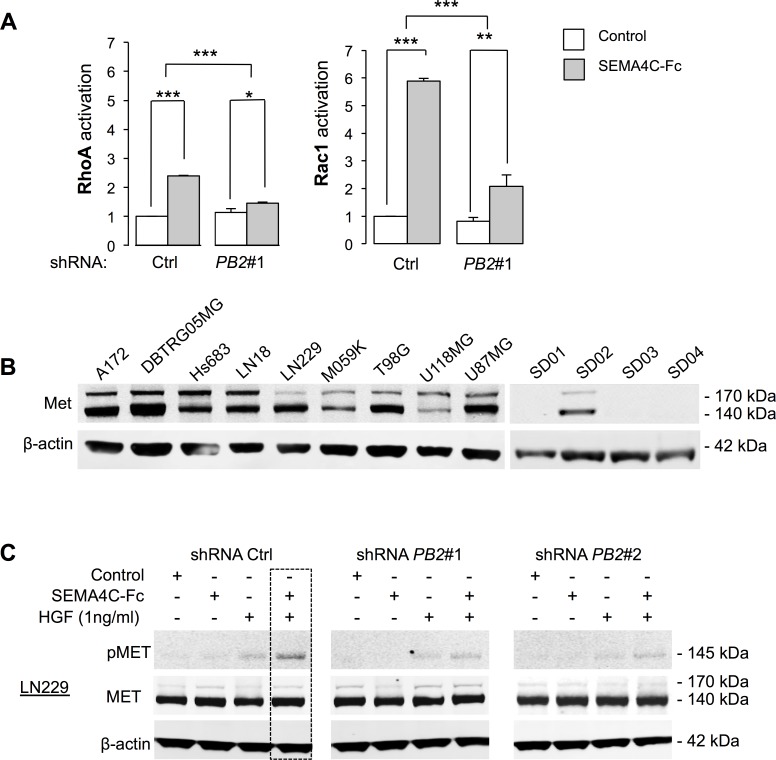
Plexin-B2 stimulation activates small GTPases RhoA and Rac1 and synergizes with HGF in Met phosphorylation A) Sema4C-Fc stimulation for 5 min activates RhoA and Rac1 in LN229 cells measured by a G-LISA Activation Assay (Cytoskeleton) (n=3). B) Western blot analysis of Met expression in ATCC glioma cell lines and GSC (the 140 kDa band represents a processed form of Met). C) Analysis of Met phosphorylation (pMet) in LN229 cells after stimulation with SEMA4C-Fc, HGF (at sub-saturating concentration of 1 ng/ml) or both shows synergy on pMet levels.

To determine downstream effectors of Plexin-B2 in glioma cells, we first investigated whether Plexin-B2 signaling activates Rho GTPases, which are mediators of invasive migration in glioma cells and known downstream effectors of Plexin-Bs in non-neuronal cell lines [24, 26]. Stimulation of LN229 cells with SEMA4C-Fc led to a 2-fold increase in activated RhoA-GTP levels and a 5-fold increase in activated Rac1-GTP levels (Fig. 5A). These effects were significantly attenuated by Plexin-B2 knockdown, indicating that Rho GTPase activation by Sema4C is transduced through a Plexin-B2 dependent pathway. Time course studies showed that activation of Rho GTPases was reached within a minute upon stimulation with SEMA4C-Fc (Fig. S6).

In cell lines of breast cancers and other non-CNS carcinomas,, Met and ErbB2, members of the RTK family, have been described as signaling partners of Plexin-B1 [16-18], and we thus investigated whether they interact with Plexin-B2 in glioma cells. We first analyzed levels of Met and ErbB2 in our collection of glioma cell lines by Western blot. Met was strongly detected in all ATCC glioma cell lines, but only in one GSC line, SD02 (Fig. 5B), whereas ErbB2 was only weakly detected in glioma cell lines (data not shown). Our subsequent investigations thus focused on Met to assess its potential interaction with Plexin-B2.

Stimulation of glioma cells with hepatocyte growth factor (HGF), the cognate Met ligand, robustly triggered Met phosphorylation in LN229, U87MG and SD02 GSC in a dose-dependent manner (Fig. S7A). Treatment with SEMA4C-Fc alone did not increase Met phosphorylation in LN229 cells, but combined stimulation with HGF (administered at a sub-saturating concentration of 1 ng/ml) resulted in synergistic Met phosphorylation, as evidenced by an approximate 50% increase in Met phosphorylation as compared to stimulation by HGF alone (Fig. 5C). The Plexin-B2 knockdown nearly abolished the synergistic effect of SEMA4C-Fc/HGF on Met phosphorylation, confirming that this effect is mediated by Plexin-B2 (Fig. 5C). Similar results were observed in U87MG and SD02 cells (Fig. S7B). It is worth mentioning that under higher concentrations of HGF (>5 ng/ml), additional SEMA4C-Fc stimulation yielded no further gain in Met phosphorylation (Fig. S7C). Together, these findings suggest that the Sema4C/HGF synergy might be biologically significant at sub-saturating concentrations of HGF.

### Plexin-B2 promotes invasive growth and vascularization of glioma *in vivo*

We next investigated the *in vivo* function of Plexin-B2 in glioma invasive migration and growth using an orthotopic xenotransplant model. For each transplant, 10^5^ cells were injected into the striatum of immunocompromised SCID mice, and after 2 weeks, brains were histologically analyzed for tumor size, tumor cell invasion, and microvascularization. Tumor tissue was readily distinguishable from host tissue by the expression of a GFP reporter from the shRNA lentiviral vector.

**Figure 6:**
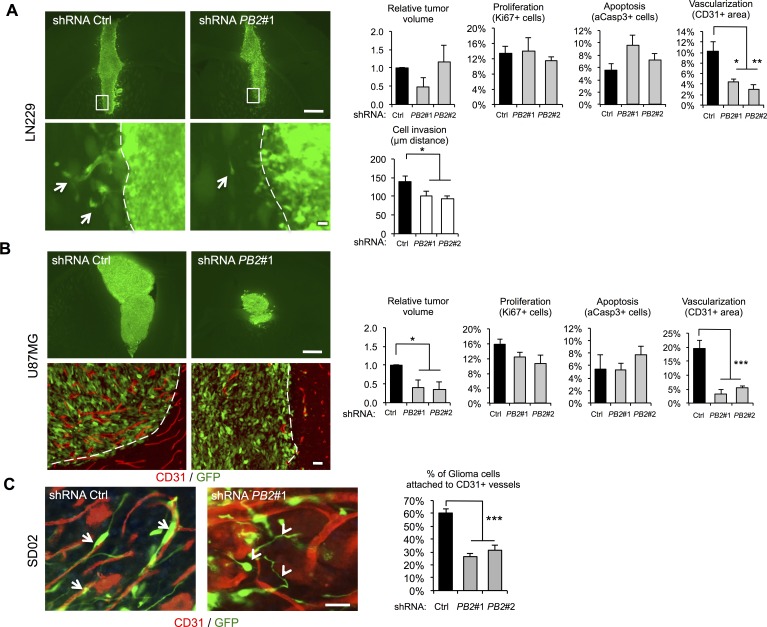
Invasive glioma growth is diminished by Plexin-B2 knockdown (A-B) Representative images and quantifications of intracranial transplants of LN229 (A) and U87MG cells (B) at 2 weeks post-transplant (n=3 transplants for each cell line). Tumor cells are labeled by GFP expression (green), and microvessels by CD31 staining (red). Arrows point to cell invasions from main tumor mass. Scale bars: 500 μm (upper panels), 25 μm (lower panels). C) Intracranial transplants of SD02 GSC at 2 weeks post-transplant show close alignment and attachment of tumor cells (GFP+, green) and microvessels (CD31+, red) in control shRNA transplants (arrows). Plexin-B2 knockdown results in misalignment and reduced percentage of infiltrating tumor cells attached to microvasculature (arrowheads). Quantification is shown on the right (n=3). Scale bar: 25 μm.

In control LN229 transplants, glioma cells displayed infiltrative growth into neighboring brain parenchyma in the form of finger-like protrusions. This invasive phenotype was significantly reduced by Plexin-B2 knockdown, thus supporting a proinvasive role of Plexin-B2 *in vivo* (Fig. 6A). Notably, Plexin-B2 knockdown resulted in no significant differences of LN229 transplants in overall tumor volume, proliferation rates, or apoptosis (Fig. 6A). Remarkably, immunostaining for the endothelial marker CD31 revealed a marked reduction in tumor vascularization in Plexin-B2 knockdown tumors (Fig. 6A).

In control U87MG transplants, by 2 weeks, highly vascularized tumor masses had expanded significantly into the host brain and individual glioma cells were closely associated with tumor microvessels (Fig. 6B), consistent with an autovascularization mode of glioma expansion along preexisting microvasculature [27]. In Plexin-B2 knockdown U87MG transplants, tumor areas were largely devoid of CD31+ microvessels and the overall tumor volumes were markedly reduced. However, no significant changes in the rates of proliferation or apoptosis in U87MG gliomas with Plexin-B2 knockdown were observed at 2 weeks after transplantation (Fig. 6B), indicating that effects of Plexin-B2 knockdown on tumor growth had occurred earlier.

Finally, we also examined SD02 transplants for their growth pattern, in particular, perivascular migration. In intracranial transplants, GSC lines proliferate slower than the ATCC lines, but disperse much wider into healthy brain tissue, which impedes quantification of the tumor bulk size at 2 week post-transplant. We thus focused our analysis on the infiltrative perivascular spreading of individual glioma cells along preexisting vasculature. In control SD02 transplants, we found that the majority of migrating cells were aligned and attached to microvessels (Fig. 6C). In Plexin-B2 knockdown SD02 transplants, however, tumor cells appeared in random orientations relative to the microvessels, and a significantly smaller fraction of tumor cells were attached to the microvessels (Fig. 6C), supporting a model that Plexin-B2 promotes perivascular migration of glioma cells.

## DISCUSSION

Our study unravels for the first time a link between Plexin-B2 and glioma invasiveness. We show that Plexin-B2 is upregulated in malignant gliomas on both transcript and protein levels. Upon stimulation by Sema4C, Plexin-B2 signaling: i) alters actin cytoskeleton and cell morphology, ii) enhances glioma cell migration, and iii) promotes glioma invasive growth, in particular, along microvasculature. Importantly, our results align with clinical data on a correlation between upregulated Plexin-B2 expression and shorter patient survival.

Among the three Plexin-B receptors, Plexin-B2 appears to be uniquely involved in glioma malignancy. Even though high immunoreactivity for Plexin-B1 has been reported in a subset of high-grade glioma [28], overall expression of Plexin-B1 or -B3 are not upregulated in gliomas, and their expression levels do not correlate with glioma grade nor patient survival. Other classes of Plexins, Plexin-As in particular, have also been implicated in regulating glioma cell migration [29-33]; whether they act in parallel of Plexin-B2 or regulate distinct aspects of glioma cell motility awaits future study.

The cognate *in vivo* ligands for Plexin-B2 in glioma remain to be determined. Sema4C appears to be the main Plexin-B2 ligand in neurodevelopment [12, 14], but the Sema4 family comprises six members that can all potentially bind to Plexin-B2. Given the importance of cell-cell interactions between glioma and stromal cells in tumor microenvironment, it will be important to define the expression patterns of distinct Sema4s in glioma and stromal cells to elucidate the molecular identity and cellular sources of Plexin-B2 ligands.

Our findings suggest a potential synergy between Plexin-B2 and the RTK Met in glioma cells, which echoes earlier findings on Plexin-B1/Met interaction in promoting tumor growth and invasion of breast cancer and other non-CNS carcinoma cells [16, 17]. Met is well known for its promigratory function in glioma cells [34], hence, Plexin-B2/Met interaction may further facilitate glioma migration. It is noteworthy that synergistic Met phosphorylation upon Sema4C/HGF costimulation only occurs at low concentrations of HGF. This implies that the Plexin-B2/Met synergy may become biologically relevant when glioma cells encounter sub-threshold levels of HGF, a situation conceivably occurring at the invasive front of gliomas. Future studies to simultaneously perturb both Plexin-B2 and Met pathways are needed to evaluate the therapeutic potential of a combinatorial treatment strategy to curb glioma growth.

Besides directly controlling tumor cell motility, Plexin-B2 may also promote invasive glioma growth by influencing cell-cell interactions between glioma and stromal cells in the tumor microenvironment. Glioma cells often display a predilection for perivascular migration along preexisting brain vasculature [5]. The basement membrane covering the microvessel surface is rich in extracellular matrix proteins, which can facilitate cellular adhesion/migration along blood vessels and also provide pro-survival signals. In a gene profiling study of brain-specific endothelial cells, Sema4C was predicted to be an endothelial-derived signal that activates pericyte-expressed Plexin-B2 [35]. It is therefore likely that endothelial/glioma cell interactions might be mediated through Sema4C/Plexin-B2 signal transduction.

In our orthotopic glioma transplant studies, vascularization of LN229 and U87MG transplants was markedly reduced with Plexin-B2 knockdown. A recent study on glioma expansion proposes an autovascularization model, in which glioma cells migrate through the perivascular space of pre-existing brain microvasculature to invade into the surrounding brain tissue [27]. Consistent with this model, the phenotype of reduced vascularization in Plexin-B2 knockdown U87MG gliomas may be attributable to stunted perivascular spreading. In addition, reverse signaling of Sema4s has been reported [36], it is thus conceivable that Plexin-B2 expressed by glioma cells may signal through Sema4C on endothelial cells to promote vascular proliferation.

Plexin-B2 knockdown resulted in smaller tumor masses of U87MG but not LN229 transplants, suggesting that LN229 glioma cells might rely less on perivascular spread but more on other processes to gain access to nutrition and pro-growth signaling. Future studies are needed to clarify the mechanisms underlying these differences. Notwithstanding, in both LN229 and U87MG glioma cells, Sema4C/Plexin-B2 signaling promotes cell migration *in vitro*, synergistic Met activation, and vascularization of tumor mass, thus underscoring generality of Plexin-B2 function for glioma progression. It is worth noting that the invasive patterns of LN229 or U87MG cells are different from one another, and do not recapitulate all aspects of infiltrative glioblastoma invasion. Future in-depth studies with patient-derived neurosphere GSC lines such as SD02 will further elucidate Plexin-B2′s role in regulating glioma malignancy.

In summary, we identified Plexin-B2 as a potential new prognostic marker for malignant glioma, and a novel drug target to curtail migratory invasion of glioma. Biologic inhibitors for Plexin-B2 activity may include soluble Plexin-B2 fusion proteins for ligand sequestration, function-blocking antibodies, or peptides blocking the binding interface [37]. The synergistic activity of Plexin-B2 and Met also provides a rationale for simultaneously targeting both pathways with combinatorial reagents. Finally, we propose a model of glioma/endothelial cell-cell interaction mediated by Sema4/Plexin-B signaling that facilitates glioma perivascular invasion. Targeting the Sema4/Plexin-B2 pathway may not only slow down glioma invasion, but also disrupt glioma vascularization, a critical step in tumor progression.

## MATERIALS AND METHODS

### Database analysis

Gene expression levels of Plexin-B and Sema4 genes were retrieved from databases NCBI/GEO (http://www.ncbi.nlm.nih.gov/geo; for datasets [20, 21]), NCI/TCGA (http://cancergenome.nih.gov), and NCI/Rembrandt (http://caintegrator.nci.nih.gov/Rembrandt). Glioblastoma subtypes of TCGA samples were obtained from reference [4].

### Cell lines and patient samples

Human glioma cell lines were obtained from ATCC resource center. Glioma stem cell lines SD01-04 were established from glioblastoma patients at UCSD and maintained under neural stem cell conditions [22]. Normal human astrocytes were purchased from Lonza. Patient samples and normal cortex were obtained from Mount Sinai Tissue Repository. Patient procedures were in accordance with protocols approved by UCSD and Mount Sinai Institutional Review Boards.

### Lentiviral shRNA knockdown

Stable knockdown of Plexin-B2 was achieved with GIPZ lentiviral vectors expressing shRNA against Plexin-B2, along with GFP and Puromycin resistance markers (Thermo Scientific, RHS4430-100986735 (shRNA PB2#1) and RHS4430-100989559 (shRNA PB2#2)). A GIPZ vector with non-targeting shRNA served as control (Thermo Scientific RHS4346). Lentiviral particles were produced with 293T cells co-transfected with GIPZ plasmid, envelope plasmid pMD2.G, and packaging plasmid psPAX2 (Addgene plasmids 12259 and 12260; deposited by Didier Trono, EPFL Lausanne). Stable GIPZ sublines were established by Puromycin (1 μg/ml) selection.

### SEMA4C-Fc stimulation of glioma cells

A human SEMA4C-Fc expression plasmid was generated by insertion of a Sema4C cDNA fragment (IMAGE Clone #40035252, Thermo Scientific) into the backbone of pEF-SEMA4D-Fc [23]. SEMA4C-Fc protein was collected as supernatant from transfected 293 cells in Opti-MEM media (Invitrogen) and concentrated by Spin-X UF column centrifugation (Corning). The supernatant of 293 cells transfected with a GFP plasmid was used as control in all stimulation experiments. SEMA4C-Fc protein concentrations were measured with fluorescent Western blot (LI-COR) by comparing SEMA-Fc with a human IgG standard (GenScript).

Glioma cells were seeded at 5 × 10^5^ cells per well in 6 well plates and cultivated for 1 day (reaching 70-80% confluence). Cells were serum-starved overnight in DMEM media with 0.2% FBS (LN229, U87MG) or growth factor-free neural stem cell media (SD02). Cells were stimulated for 1 hour in serum-starved media with addition of control supernatant, 100 ng/ml SEMA4C-Fc, 1 ng/ml HGF (Peprotech), or both.

### Immunolabeling

Primary antibodies: anti-activated-caspase 3 (Abcam 2302), anti-beta-actin (Sigma A1978), anti-CD31 (BD Biosciences 553370), anti-Ki67 (Abcam 15580), anti-Met (D1C2; Cell Signaling 8198), anti-phospho-Met (Tyr1234/1235; Cell Signaling 3077), anti-Plexin-B1 (A-8; Santa Cruz sc-28372), anti-Plexin-B2 (R&D AF5329), anti-Plexin-B3 (R&D AF4958), anti-Sema4C (LSBio LS-C168955), anti-Vinculin (Sigma V9131).

For Western blotting, protein lysates were prepared with RIPA buffer (Sigma) containing protease and phosphatase inhibitors. Secondary antibodies IRDye 680 or 800 (LI-COR) were used for blot detection with Odyssey Infrared Imaging System (LI-COR). For immunofluorescence labeling of cells and tissue sections, Alexa-labeled secondary antibodies (Jackson ImmunoResearch) and DAPI (Invitrogen) nuclear staining were used.

Tissue microarray analysis was performed with arrays T175 and GL803a (US Biomax) containing cores from glioblastoma (n=34), astrocytoma (n=21), oligodendroglioma (n=10), oligo-astrocytoma (n=4), and normal brain (n=7). Tissues were subjected to heat-induced epitope retrieval with Basic Antigen Retrieval Reagent (R&D), stained for immunosignals with DAB kit (R&D), and counterstained with Gill's #2 hematoxylin.

### Scratch wound migration assay

Glioma cells were grown to confluence, serum-starved (DMEM with 0.5% FBS) for 24 hours, and a strip of cells was scratched at the center of the dish and media was replaced with fresh starvation medium containing control supernatant or 100 ng/ml SEMA4C-Fc. The area of the scratch was measured in Photoshop (Adobe Systems) at 0 and 24 hours to determine wound closure rates.

### Intracranial transplants

Animal procedures were performed in accordance with protocols approved by Mount Sinai IACUC committee. 10^5^ glioma cells in 1 μl cell suspension were stereotactically injected into the striatum (Paxinos coordinates: +2 mm lateral, -0.5 mm AP, -2.5 mm vertical) of adult immunocompromised ICR-SCID male mice (IcrTac:ICR-*Prkdc*^scid^; Taconic Farms). After 14 days, mice were perfused with 4% paraformaldehyde/PBS and brains were prepared for histological analysis.

Tumor volume was calculated from the size of GFP+ areas on consecutive sections (Photoshop). For cell invasion measurements, three sections with the largest GFP+ areas were chosen from each tumor, and the invasion distance of GFP+ cells was defined as the distance between individual cells and border of main tumor mass.

### Statistical Analyses

One-way analysis of variance followed by Tukey's post-hoc test was performed using Prism statistical software (GraphPad). All bar graphs represent mean and error bars represent standard error of the mean. *, *p*<0.05; **, *p*<0.01; ***, *p*<0.001.

## SUPPLEMENTARY MATERIAL, FIGURES, TABLES


