# Supplementary material for: Plexin-B2 promotes invasive growth of malignant glioma
Source: Oncotarget. 2015 Jan 31;6(9):7293–304. doi: 10.18632/oncotarget.3421 (PMC4466685; doi:10.18632/oncotarget.3421)
Supplement: Supplementary file 1 [file oncotarget-06-7293-s001.pdf]

Plexin-B2 promotes invasive growth of malignant glioma

Supplementary Material

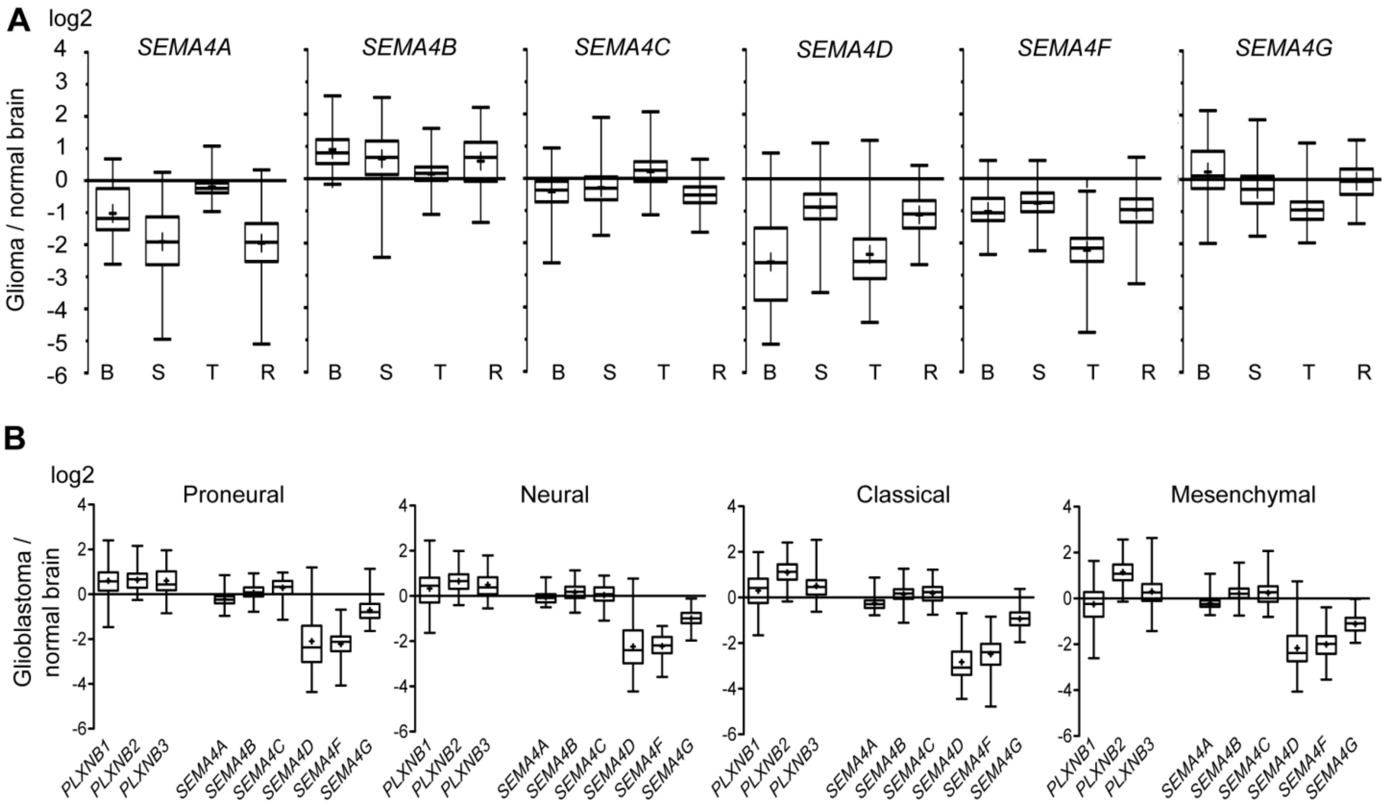

Supplementary Figure S1. Sema4 expression in glioma and Plexin-B2 expression in glioblastoma subtypes. A) Relative Sema4 gene expressions in glioma vs. normal brain in microarray data of four patient studies (B, Bredel et al. (n=49); S, Sun et al. (n=49); T, TCGA (n=424); R, Rembrandt (n=454)). Whiskers represent top and bottom quartiles. Sema4B, 4C, and 4G expression levels appeared unchanged or moderately increased, while other Sema4s were downregulated in glioma. B) Relative Plexin-B and Sema4 gene expression in molecular subtypes of glioblastoma (TCGA glioblastoma data; proneural, n=113; neural, n=70; classical, n=112; mesenchymal, n=128).

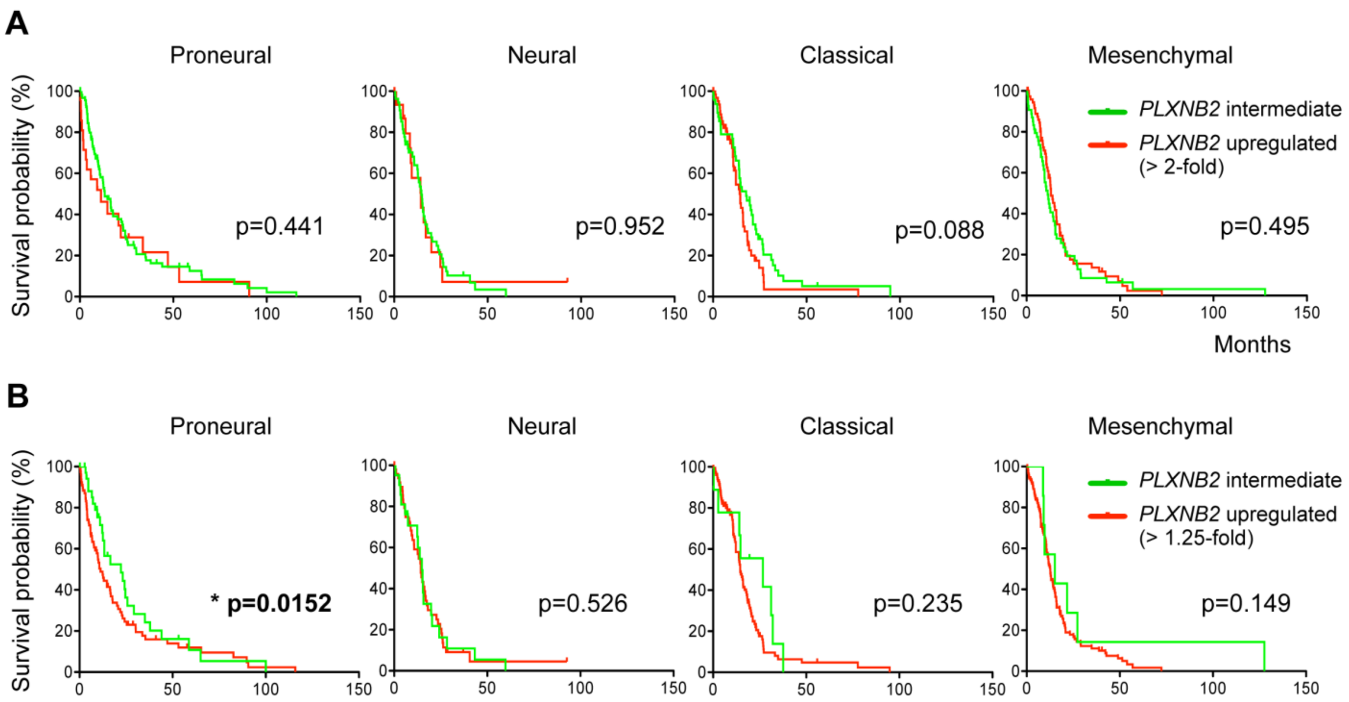

Supplementary Figure S2. Survival probabilities of patients of TCGA glioblastoma subtypes in dependence of Plexin-B2 expression.

A) Survival probabilities for patients with upregulated Plexin-B2 expression (> 2-fold above normal level) in TCGA molecular subtypes of glioblastoma (proneural, n=113; neural, n=70; classical, n=112; mesenchymal, n=128)). Log-rank p values are indicated in the graph. B) Survival probabilities for patients were recalculated using a > 1.25-fold above normal level threshold for upregulated Plexin-B2 expression. Note statistically significant shorter median survival for proneural subtype with upregulated Plexin-B2 expression.

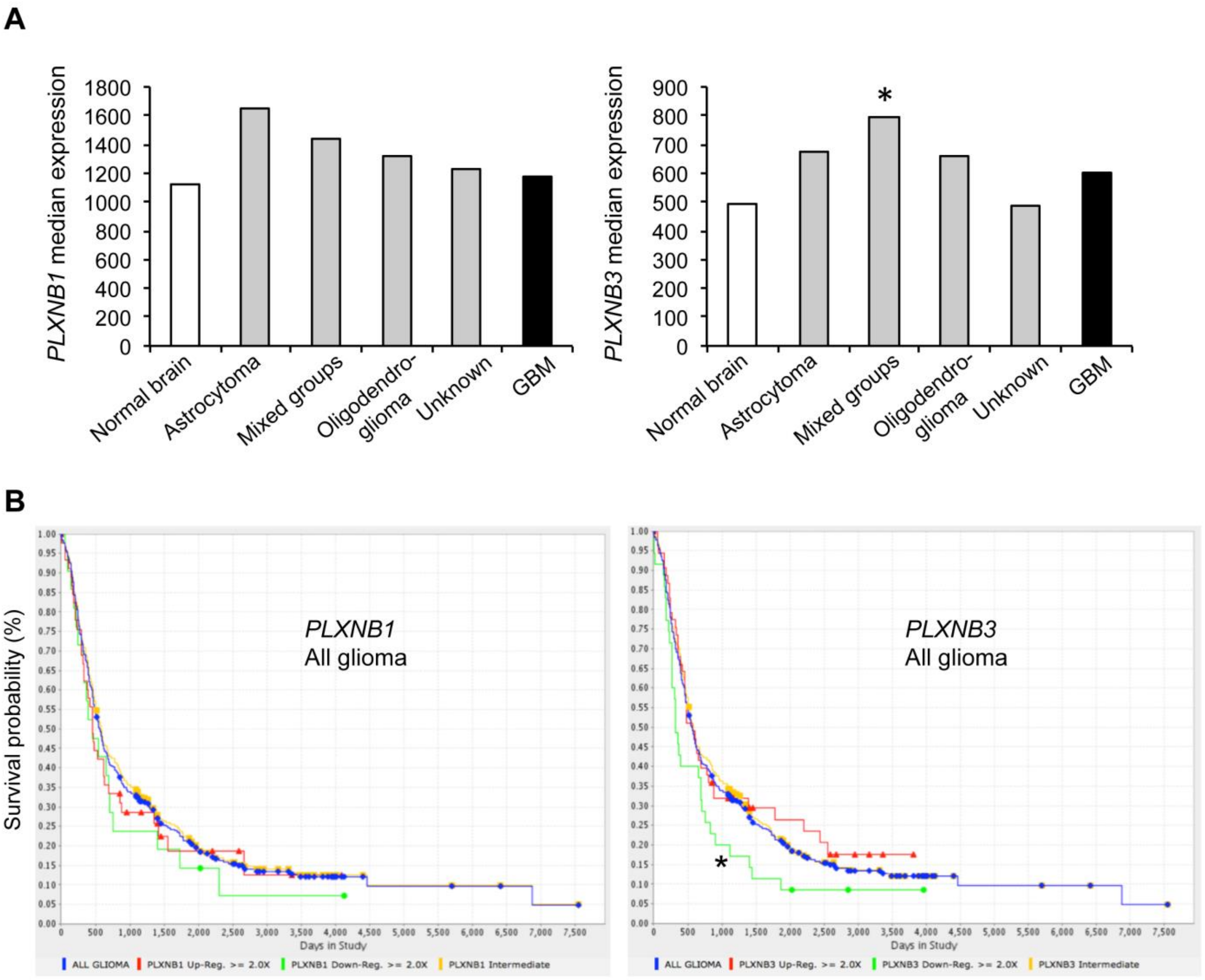

Supplementary Figure S3. Plexin-B1 and -B3 expression in glioma and correlation with survival.
A) Median expression level of Plexin-B1 and -B3 in normal brain and different glioma types (data from NCI/Rembrandt platform; probesets 215807_s_at and 205957_at). No significant differences between glioma types and normal brain were found, except an increase of Plexin-B3 in the mixed group (p<0.03). B) Kaplan-Meier survival analysis for glioma patients with different expression levels of Plexin-B1 or -B3 as displayed by the NCI/Rembrandt platform. Down-regulated Plexin-B3 expression corresponds with shorter survival for all glioma patients (p<0.04).

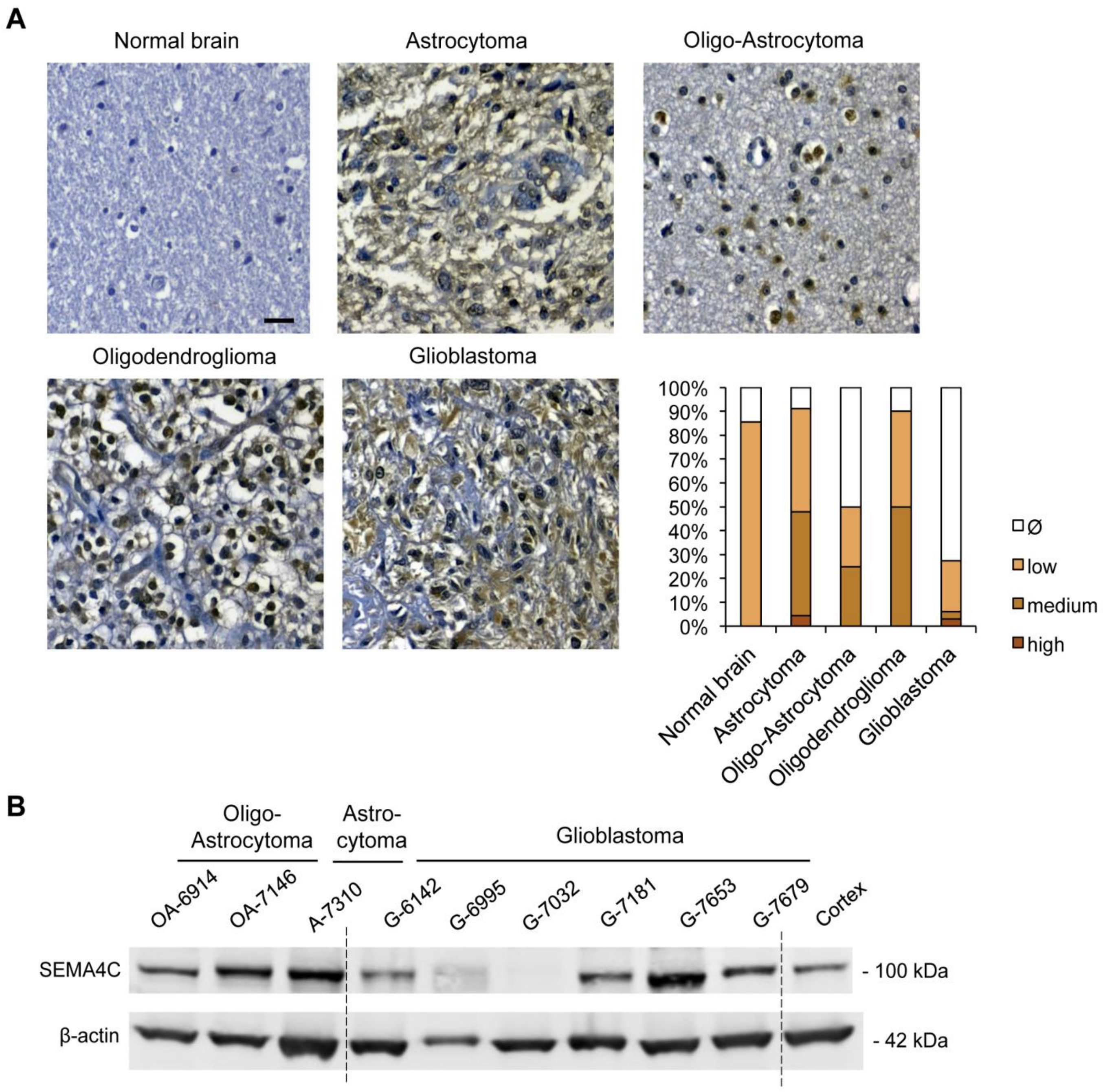

Supplementary Figure S4. SEMA4C is expressed in gliomas.

A) Representative images of DAB immunolabeling of Plexin-B2 protein expression in normal brain and glioma tissues (n=69). Bar graph summarizes scored immunosignal intensities in different glioma types (low, average SEMA4C immunointensity in normal brain; Ø, no signal detected). Scale bar: 20 μm. B) Western blot analysis of Sema4C protein in lysates from tumor samples.

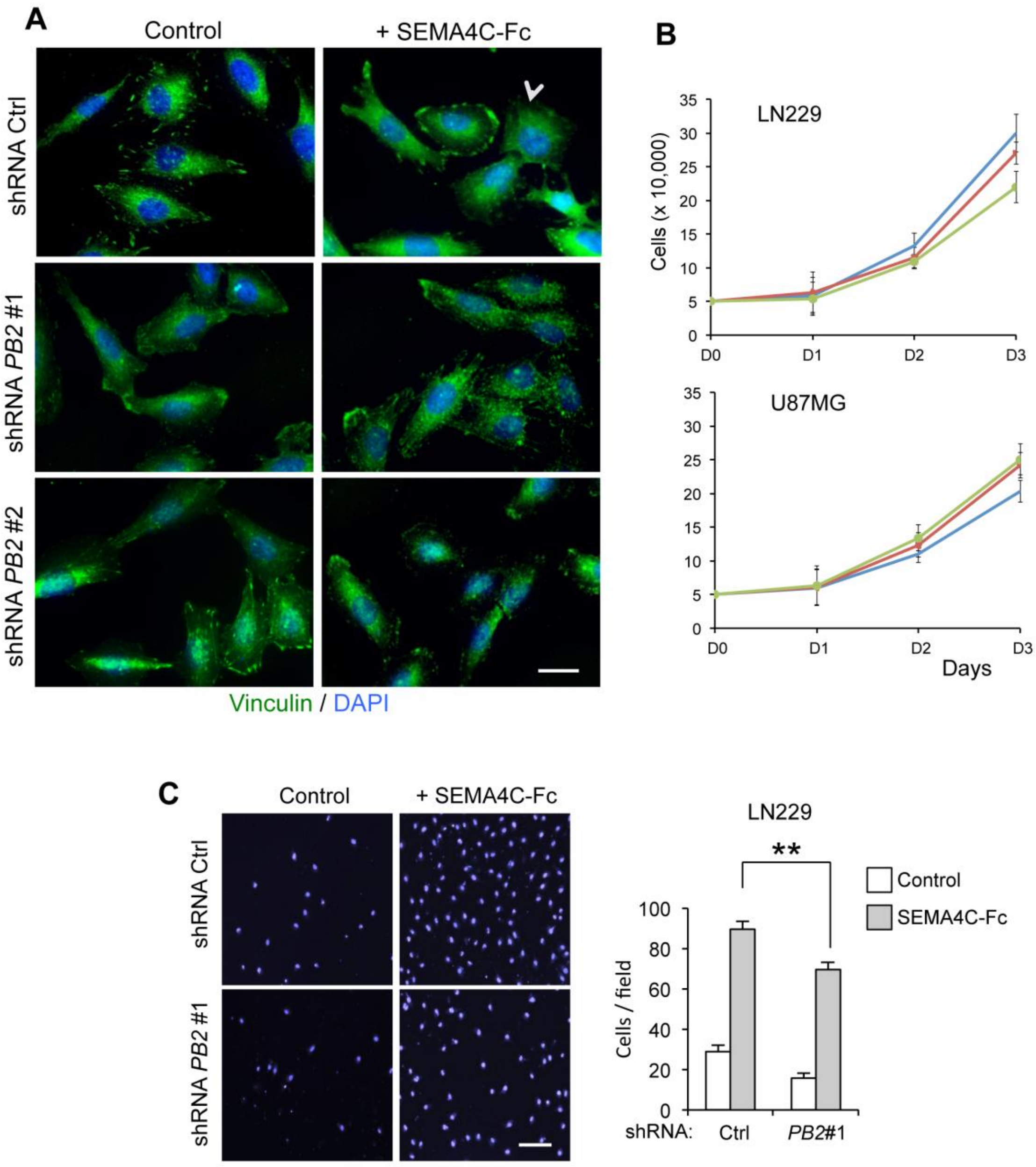

Supplementary Figure S5. Effect of SEMA4C-Fc stimulation in glioma cells.

A) LN229 cells with or without Plexin-B2 shRNA knockdown were stimulated with SEMA4C-Fc or control supernatant for 5 min. Staining for the focal adhesion complex component vinculin revealed loss of focal adhesions following SEMA4C-Fc stimulation. These effects were reduced in Plexin-B2 shRNA knockdown lines. Scale bar: 20 µm. B) Growth curves showed no significant differences in the proliferation rates between control and Plexin-B2 shRNA lines. Glioma cells were seeded at 50,000 cells/well in triplicate 6-well tissue culture plates and cell numbers were measured over the course of 3 days. C) Matrigel transwell invasion assay with LN229 cells. Upper compartments of Matrigel-coated chambers (BD BioScience) were seeded with 5 x 10^4 cells in DMEM with 0.5% FBS. Lower compartments were filled with media containing 0.5% FBS and 100 ng/ml SEMA4C-Fc or control supernatant. Invasive migration of cells to lower side of membrane was visualized after 24 hours by DAPI-staining. The proinvasion effect of SEMA4C-Fc was decreased but not completely abrogated by Plexin-B2 knockdown (n=4; $p<0.01$), suggesting possible residual Plexin-B2 activity under knockdown conditions or other compensatory mechanisms in this assay. Scale bar: 50 µm.

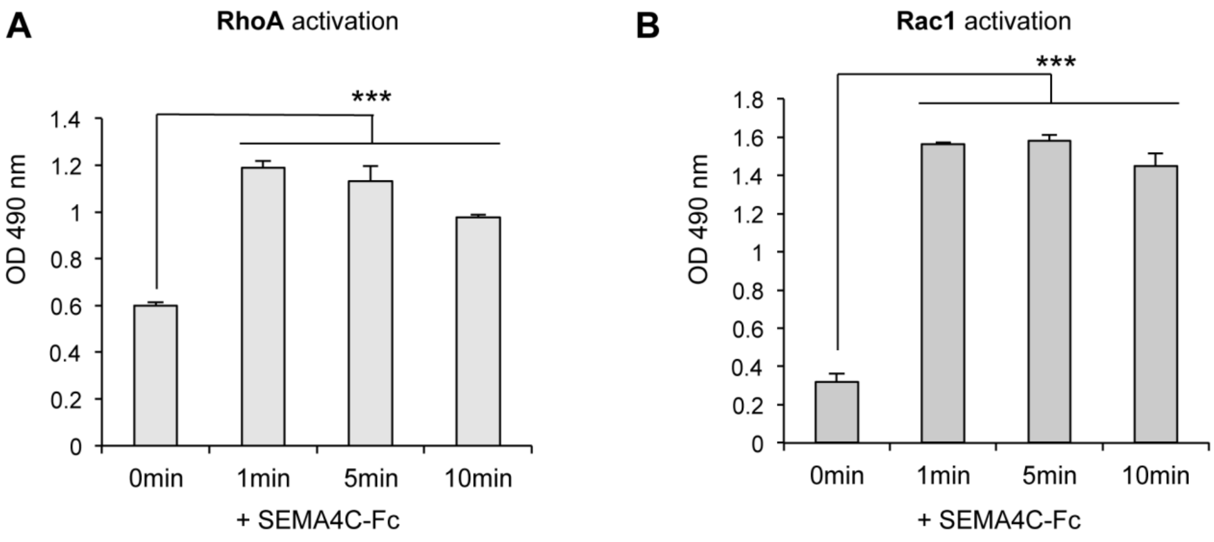

Supplementary Figure S6. Time course of RhoA and Rac1 activation in LN229 cells by SEMA4C-Fc.
A, B) LN229 glioma cells were stimulated with SEMA4C-Fc for 1 to 10 min, and activation of RhoA (A) and Rac1 (B) were measured with G-LISA assays. LN229 cells showed sustained increase in RhoA-GTP and Rac1-GTP after 1 min stimulation until 10 min.

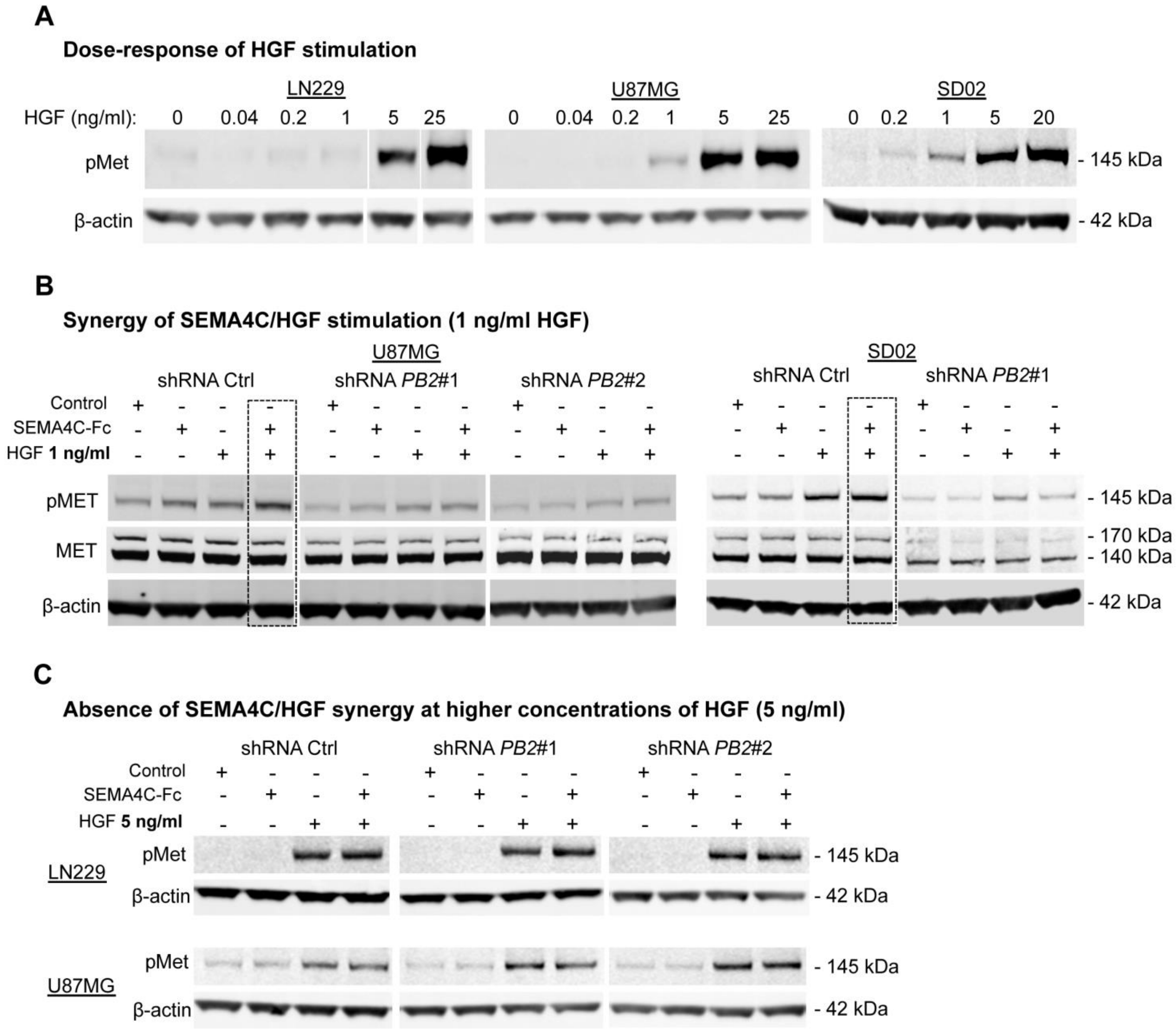

Supplementary Figure S7. Dependence of Met phosphorylation on HGF ligand concentration.
A) Detection of phospho-Met (pMet) in LN229, U87MG and SD02 GSC cells that were stimulated with increasing concentrations of HGF for 60 min. The 1 ng/ml HGF concentration was chosen for further costimulation experiments with SEMA4C-Fc. B) Analysis of Met phosphorylation in U87MG and SD02 cells by stimulation with SEMA4C-Fc, HGF (1 ng/ml) or both showed synergy of Met phosphorylation by combined Sema4C-Fc/HGF stimulation. Synergistic effects were decreased in cells with Plexin-B2 shRNA knockdown. C) Western blot analysis of pMet in glioma cells after stimulation with a high concentration of HGF (5 ng/ml) showed no detectable synergistic SEMA4C-Fc/HGF activity in Met phosphorylation.

Supplementary Table S1.

Median overall survival in Rembrandt glioma patient cohorts, listed by Plexin-B2 gene expression level and glioma type. Data retrieved from http://caintegrator.nci.nih.gov/rembrandt. Log-rank p-value signifies difference of survival between upregulated (expression >2-fold above normal) and intermediate cohorts.

	***PLXNB2* intermediate**		***PLXNB2* upregulated**		
	patients (n)	median survival (months)	patients (n)	median survival (months)	*Log rank p-Value*
**All glioma**	100	32.2	243	16.0	3.94 E-5
**Astrocytoma**	40	58.2	65	23.0	0.00576
**Oligodendroglioma**	20	29.3	32	24.9	0.428
**Glioblastoma**	37	17.5	144	13.95	0.662
